# The Influence of Emotional Awareness on Time Perception: Evidence From Event-Related Potentials

**DOI:** 10.3389/fpsyg.2021.704510

**Published:** 2021-12-24

**Authors:** Jia Ma, Jiamei Lu, Xu Li

**Affiliations:** Department of Psychology, Shanghai Normal University, Shanghai, China

**Keywords:** overestimated, emotional stimulus, arousal mechanism, emotional awareness, time perception

## Abstract

Prior studies found that participants overestimated both negative and positive emotional stimuli, compared with neutral emotion. This phenomenon can be explained by the “arousal mechanism.” Participants demonstrated individual differences in emotion perception. In other words, high emotional awareness resulted in high emotional arousal, and vice versa. This study extended existing findings by exploring the influence of emotional awareness on time perception in a temporal generalization task, while recording electroencephalographic (EEG) signals. The findings revealed that in the positive emotion condition, the high emotional awareness group made more overestimations, compared with the low emotional awareness group. However, no difference was observed in the neutral or negative emotion conditions. Moreover, the event-related potential (ERP) results showed that in the positive emotion condition, the high awareness group elicited larger vertex positive potential (VPP) amplitudes, compared with that of the low awareness group. However, no such differences were observed in the neutral and negative emotion conditions. Moreover, the contingent negative variation (CNV) (200–300, 300–490 ms) component showed that in the positive emotion, the amplitudes of the high awareness group were larger than that of the low awareness group; however, they did not show differences in the neutral condition. The findings of this study suggest that high emotional awareness produces higher physiological arousal; moreover, when participants were required to estimate the time duration of emotional pictures, they tended to make higher time overestimation. Thus, our results support the relationship between emotional awareness and time perception.

## Introduction

Time perception differs from the accuracy of the clock, and it is a subjective temporal experience, which is influenced by emotion. A number of studies showed that emotional arousal is an important influencing factor of time perception (e.g., Droit-Volet et al., 2013; Fayolle et al., 2015). For example, participants in (Gil and Droit-Volet, 2012) study verbally estimated the duration of emotional pictures (i.e., neutral, disgust, and sadness). The same discrete emotion varied in arousal level (high/low arousal). The results of their study showed a lengthening effect on the time perception of the emotional picture, thus indicating that this effect increased with the arousal level. Studies using different emotional materials or experimental paradigms reached the same conclusion. That is, within the time perception of 2 s, the participants made a higher time overestimation when in high emotional arousal (Droit-Volet and Meck, 2007; Gan et al., 2009a; Jia et al., 2015). Furthermore, the emotion-based time overestimation was caused by the arousal-based mechanism, which involves the activation of an internal clock system. The internal clock modes hold that perceived duration is based on the number of pulses collected in the pacemaker; thus, the more pulses accumulated, the longer the perceived duration (Treisman, 1963; Schwarz et al., 2013; Droit-Volet and Gil, 2016; Cui et al., 2018). Emotional arousal increases the speed of the pacemaker; thus, emotional events are overestimated, compared with their neutral events.

Previous studies have proved the impact of emotional stimulus' arousal on time perception (Smith et al., 2011; Van and Balsam, 2014; Wang et al., 2016). Moreover, individual physiological differences have been found for the same type of emotional arousal (Zhang and Lu, 2013; Zhang et al., 2016). Thus, individual differences in physiological arousal may influence time perception.

Prior studies have found that individuals with high emotional awareness are more likely to produce high physiological arousal, whereas those with low emotional awareness produce low arousal (Zhang and Lu, 2013; Wang et al., 2015; Zhang et al., 2016). Emotional awareness refers to the ability to recognize and describe one's own and also others' emotions (Lane and Schwartz, 1987; Zhang and Lu, 2013; Wang et al., 2015); moreover, it is closely related to physiological arousal. Zhang et al. (2016) used blood volume pulse (BVP) and resistance from skin conductance (SCR) as emotional arousal indexes to explore the relationship between emotional awareness and emotional arousal. Their results confirmed that in the emotion conditions (positive and negative), the rate of physiological change (i.e., BVP, SCR) in the high emotional awareness group was higher, compared with that of the low emotional awareness group. Thus, their results revealed that high awareness resulted in higher emotional arousal. Their study also used path analysis to explore the mechanism from stimuli awareness to emotional arousal: when the emotional expression was presented to participants, their awareness of emotion led to unconscious imitation of the expressions. Moreover, the unconscious imitation often occurred with physiological arousal, which caused them to produce the corresponding emotion (Zhang and Lu, 2013; Zhang et al., 2016). Therefore, in the same discrete emotional stimulus, high awareness resulted in high mimicry with high emotional arousal; however, the opposite was true for low emotional awareness.

This study aimed to explore the effects of emotional awareness on time perception. In particular, we aimed to examine the neural mechanisms underlying the effects of emotional awareness on time perception using the event-related potential (ERP) technique. The neural mechanisms were primarily analyzed by the vertex positive potential (VPP) and contingent negative variation (CNV) components.

Vertex positive potential is differently modulated by emotional and neutral faces; it is a positive deflection detected at the frontocentral electrode, with peak latency between 140 and 200 ms (Williams et al., 2006; Luo et al., 2010; Zhang et al., 2013). VPP is sensitive to facial expressions in the rapid serial visual presentation paradigm (Luo et al., 2010; Zhang et al., 2013; Wang et al., 2015). Its amplitude is influenced by emotion valence, with the amplitude of the emotional stimuli which is larger than that of neutral stimuli (Luo et al., 2010). Differences in awareness levels are mainly reflected in the recognition and judgment of emotional stimuli. Prior studies have found that participants with high emotional awareness responded faster and more accurately to emotional expressions, compared with those with low awareness (Wang et al., 2015; Zhang et al., 2016). Therefore, we chose VPP components to explore the difference between high and low emotional awareness groups.

The CNV component has been suggested to reflect the accumulation process (Macar and Vidal, 2009) in pacemaker–accumulator models of interval timing [e.g., Treisman, 1963; Gibbon et al., 1984; Meck, 1996; Wearden, 1999; Meck and Benson, 2002; Taatgen et al., 2007]. It covers the median frontocentral area (FCz) and initiates ~250 ms poststimulus (Pfeuty et al., 2005, 2008; Zhang et al., 2006; Pouthas et al., 2015). According to Birbaumer et al. (1990), slow potential changes of negative polarity recorded over the scalp reflect neuronal activation in the underlying cortical layers. An increase in CNV amplitude indicates increased neuronal activation. In accumulator-based models of timing, when one target interval is to be processed, increased neuronal activation in the structures that subtend temporal encoding should be associated with longer estimates of the interval. It reflects an increase in the number of units that accumulate during the interval. Research has suggested that CNV amplitude may represent an index of temporal encoding as achieved by an accumulator mechanism: larger CNV amplitudes correspond to overestimation of time (Macar and Vidal, 2004; Olofsson et al., 2008). Thus, we used the CNV to explore the difference between the effects of high and low emotional awareness on time perception.

This study hypothesized that the high awareness group would make more time overestimation of emotional condition (positive/negative), compared with the low awareness group; however, no differences would be observed in the neutral condition. Furthermore, we generated the following hypotheses related to the ERP result are as follows: (1) for the VPP component, the emotional (positive/negative) amplitude of the high awareness group would be significantly larger than that of the low awareness group; (2) for the CNV component, the emotional amplitude of the high awareness group would be significantly larger than that of the low awareness group. Moreover, for both VPP and CNV, there would be no differences between the high and low awareness groups in the neutral condition.

## Methods

### Participants

First, we estimated our sample size with the effect size of 0.25 and power of 0.8 (see Cohen, 1988) using G^*^power (Faul et al., 2007). We aimed for a sample size of a minimum 18 participants. Then, we chose the high and low emotional awareness group participants using the Levels of Emotional Awareness Scale (LEAS; Lane and Schwartz, 1987; Lane, 2000; Wang et al., 2015). Eighty college students were selected through random sampling from the Shanghai Normal University, who then completed the LEAS. Participants who scored in the first 27% and last 27% were ranked as the high awareness group and low awareness group, respectively (Wang et al., 2015; Zhang et al., 2016). Hence, 45 right-handed volunteers were selected to enroll in the ERP experiment. Then, we excluded three participants whose electroencephalographic (EEG) signal showed excessive artifacts. The final sample include 42 participants, aged 22–24 years [mean (*M*) = 22.24, standard deviation (SD) = 0.69], with 21 participants each in the high (10 men, 11 women) and low awareness groups (11 men, 10 women). The LEAS score of the high awareness group (*M* = 2.88, *SD* = 0.17, *n* = 21) was significantly higher than that of the low awareness group (*M* =1.94, *SD* = 0.44, *n* = 21) [*t*_(38)_ = 9.06, *p* < 0.001, *D* = 0.71]. All of them had normal or corrected-to-normal vision and also no neurological or psychiatric disorders.

The study protocol was approved by the Ethical Committee of the School of Psychology at Shanghai Normal University. Written informed consent was obtained prior to the experiment onset, and all participants received monetary compensation after completing the experiment.

### Stimuli

The stimuli used for the representation of standard duration were a gray oval. The emotional stimuli were three types of facial pictures (neutral, negative, and positive), selected from the standard Chinese Facial Affective Picture System (CFAPS; Bai et al., 2005). A total of 90 pictures were selected: 30 neutral, 30 negative (10 sad, 10 angry, and 10 fear), and 30 positive (happy). Each discrete emotional expression was equally represented by typically male and female faces. Each picture was previously assessed for valence and arousal using a nine-point scale with a large sample of Chinese participants in a previous survey (valence: positive = 6.53, negative = 2.19, neutral = 4.11; arousal: positive = 5.95, negative = 5.88, neutral = 3.28).

Stimuli did not show hairs, with merely their interior characteristics being retained. Each picture was cropped into an elliptical shape using Adobe Photoshop 8.0. All stimuli were matched in terms of luminance and size (visual angle: 6.57 × 6.861°), and the screen resolution was 72 pixels per inch. All stimuli were displayed at the center of the screen (17-inch).

### Experimental Procedure

**Figure 1:**
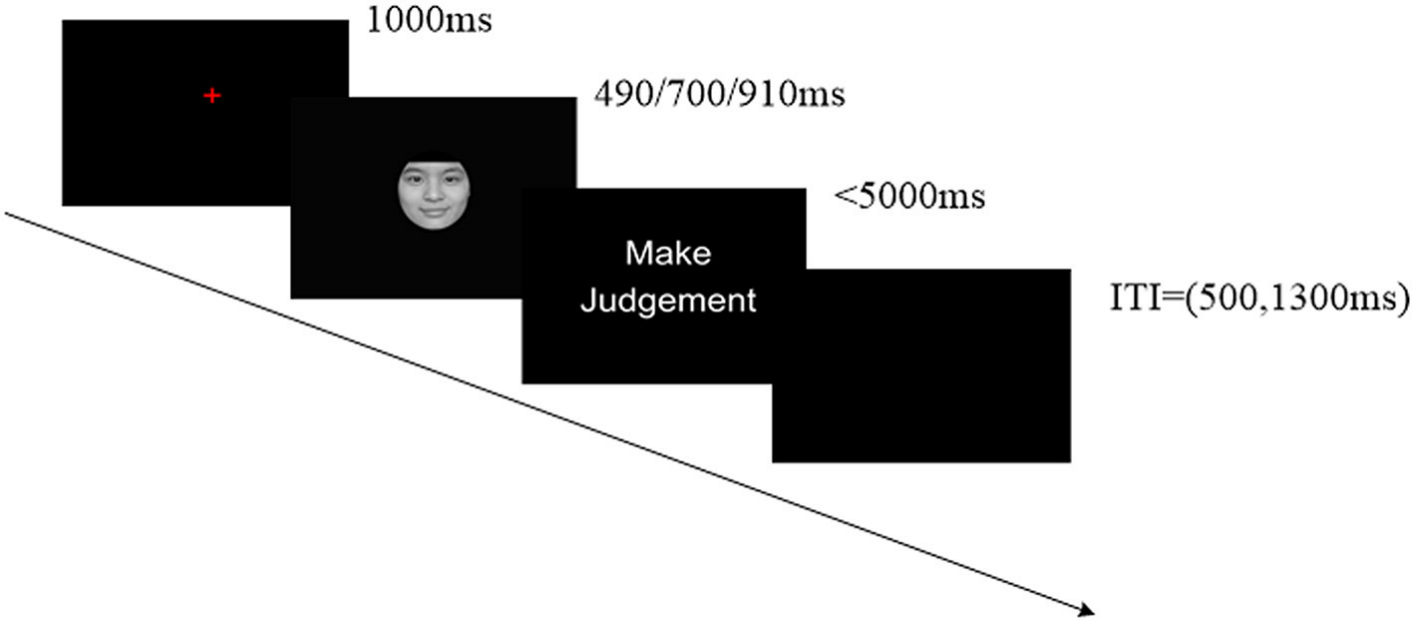
Example of one trial in the test phase. Participants were asked to focus on the crosshairs for 1,000 ms before being presented with a stimulus for 490, 700, or 910 ms; they were then asked to make a judgment within 5,000 ms. Individual trials were separated by a 500 or 1,300 ms intertrial interval (ITI). We recorded EEG data for the emotional stimulus presentation during the test phase. Human images reproduced from Chinese Facial Affective Picture System (CFAPS) with permission.

The experiment was administrated in a quiet chamber. Further, the participants were at a distance of ~50 cm from the computer monitor. We recorded the participants' responses using the E-Prime software (Psychology Software Tools Inc., Pittsburgh, USA). Participants performed a temporal generalization task (Gan et al., 2009b), which was composed of two successive phases, including learning and testing. In the learning phase, participants were shown the “standard” stimulus duration (700 ms) 10 times, which was represented by the gray oval. In the testing phase, the participants were required to judge whether the face was presented in shorter, longer, or equal durations compared with the “standard” stimulus by pressing the “F,” “SPACE,” or “J” key on the keyboard correspondingly, within 5,000 ms. The left–right response position was counterbalanced across participants. Every participant performed 360 trials, and each discrete emotion was tested 40 times at every duration (i.e., 490, 700, and 910 ms) in a random order. There was a blank interval between stimuli in each trial (randomly chosen between 500 and 1,300 ms). After every 90 trials, the participants were given a break and the “standard” duration was presented 10 times once again to prevent the participants from forgetting it. A trial in the testing phase is displayed in Figure 1.

### Behavioral Recordings and Data Analysis

In this study, the “overestimation response ratio” and “underestimation response ratio” were the dependent variables in the behavioral analysis (Gan et al., 2009a; Mella et al., 2011). Emotional awareness and emotion valence acted as independent variables in a mixed repeated measures analysis of variance (rmANOVA) with the between-factor awareness group (2: high/low) and the within-factor emotion valence (3: neutral/positive/negative). Participants' key response was used to record their time estimation (correct/overestimation/underestimation). The behavior results are divided into correct response, time underestimation, and time overestimation; the time underestimation and time overestimation are actually a wrong response. If a participant judged the duration of 490 ms to be equal to or longer than the “standard” stimulus duration (700 ms), it was considered an overestimation of time. However, if they judged it as shorter, it was considered the correct response. If the participants judged the 910 ms duration to be equal to or shorter than 700 ms, it was considered an underestimation, whereas if they judged it as “longer,” it was considered a correct response. For the 700 ms condition, “shorter” was considered an underestimation, “equal” was considered a correct response, and “longer” was considered an overestimation of time.

The “overestimation respond ratio” refers to the ratio of overestimation response trials to the total trials. For example, the positive emotion in the 490 ms condition had 40 trials; if a participant's time overestimation was 20 trials, their overestimation response ratio would be 0.5 (20/40). The statistical method of “underestimation respond ratio” is the same.

### ERP Recordings and Data Analysis

The EEG was recorded from 64 scalp sites using Ag/AgCI mounted in an elastic cap (NeuroScan Inc., EI Paso, Texas, USA). The EEG and electrooculography were amplified using a direct current (0.05 Hz) with ~100 Hz bandpass and continuously sampled at 500 Hz/channel. All interelectrode impedance was maintained below 5 kΩ. ERP averages were computed offline; trials with artifacts were rejected with a criterion of ± 80 μV. ERP waveforms were time locked to the onset of the stimulus, and the average epoch was 1,000 ms, including a 200 ms prestimulus baseline. Recording electrodes were referenced to the left mastoid and recalculated offline to an average mastoids reference. The vertical electrooculogram signal was measured both above and below the left eye, whereas horizontal electrooculogram signal was measured on both sides at the external canthi. Eye-blink artifacts were rejected automatically using vertical ocular correction. All channels were filtered at 0.1–30 Hz offline. Incorrect response trials were removed.

Similar to previous studies (Macar and Vidal, 2004; Pfeuty et al., 2005, 2008; Luo et al., 2010; Wang et al., 2015), the amplitudes of the early components of VPP and the middle-late stage of CNV components were computed. VPP (150–200 ms) average amplitudes were measured in the time window of the frontocentral sites (F1, FZ, F2; Luo et al., 2010; Wang et al., 2015). For CNV components (200–300, 300–490 ms), FCz was used for statistical analysis (Macar and Vidal, 2004; Pfeuty et al., 2005, 2008; Zhang et al., 2006). Average amplitudes for each component were measured by a mixed rmANOVA, with emotional awareness (high/low) as the between-subjects factor and emotional valence (neutral/positive/negative) and electrodes (VPP: F1, FZ, F2; CNV: FCz) as within-subject factors. The Greenhouse–Geisser epsilon correction was applied to adjust the degrees of freedom of the F ratios, and multiple comparisons were conducted using the Bonferroni method.

## Results

### Behavioral Results

For the overestimation response ratio as dependent variable, a mixed rmANOVA with the between-factor awareness group (2: high/low) and the within-factor emotion valence (3: neutral/positive/negative) revealed a main effect of emotion valence, *F*_(2,80)_ = 13.16, *p* < 0.001, η^2^= 0.25. *Post hoc* analysis with Bonferroni correction showed that time overestimation of the positive condition (0.184 ± 0.01) was greater than the neutral condition (0.166 ± 0.01), *p* = 0.002, whereas that of the negative emotion condition (0.190 ± 0.01) was greater than the neutral condition, *p* < 0.001. There were no significant differences between the positive and negative emotion conditions (*p* = 0.51). The interaction between emotion valence and awareness group was significant, *F*_(2,80)_ = 8.58, *p* < 0.001, η^2^ = 0.18. Simple-effect analysis showed that in the positive emotion condition, the high awareness group overestimated time more than the low awareness group, *F*_(1,40)_ = 4.86, *p* = 0.03, η^2^ = 0.11. There were no significant differences between high and low awareness groups in the neutral or negative emotion conditions (*ps* ≥ 0.98); emotional awareness had no main effect (*p* = 0.41).

For the underestimation response ratio as the dependent variable, a mixed rmANOVA with the between-factor awareness group (2: high/low) and the within-factor emotion valence (3: neutral/positive/negative) did not reveal either significant main effect (*p*s ≥ 0.36) or interactive effects (*p* = 0.99).

**Figure 2:**
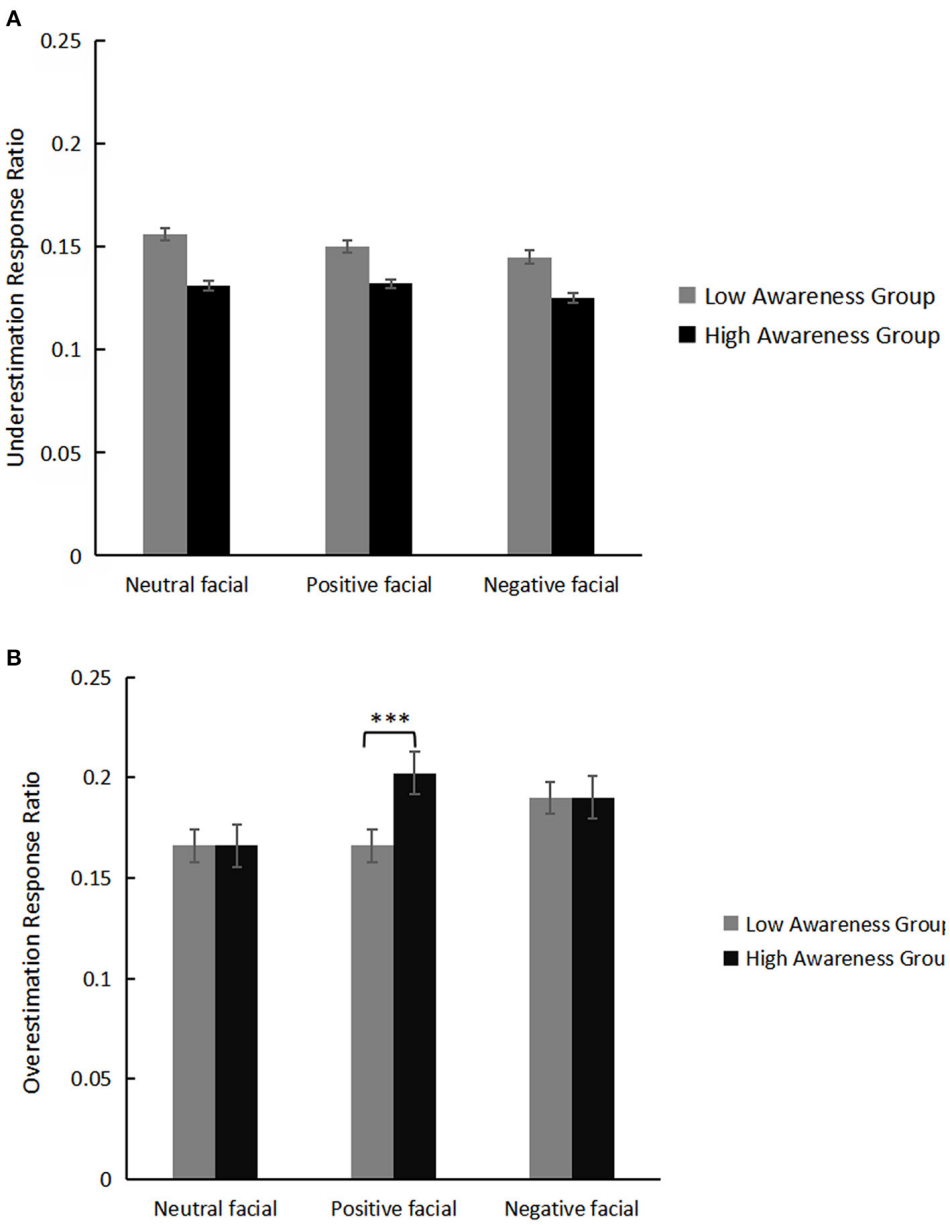
Means and standard error of underestimation response ration **(A)** and overestimation response ratio **(B)** for the high and low awareness groups. “***” represents the *p* < 0.001.

Means and *SE* of overestimation and underestimation response ratios for the two groups are displayed in Table 1. Behavior results of the time underestimation and time overestimation between the two groups are displayed in Figure 2.

**Table 1:** Means and standard error of overestimation and underestimation response ratio for the high and low awareness groups.

	**Underestimation (*****M*** **±*****SE*****)**		**Overestimation (*****M*** **±*****SE*****)**	
	**Low awareness group**	**High awareness group**	**Low awareness group**	**High awareness group**
Neutral facial expression	0.156 ± 0.01	0.131 ± 0.01	0.166 ± 0.01	0.166 ± 0.01
Positive facial expression	0.150 ± 0.01	0.133 ± 0.01	0.166 ± 0.01	0.202 ± 0.01
Negative facial expression	0.145 ± 0.01	0.125 ± 0.01	0.190 ± 0.01	0.190 ± 0.01

### ERP Results

#### VPP

**Figure 3:**
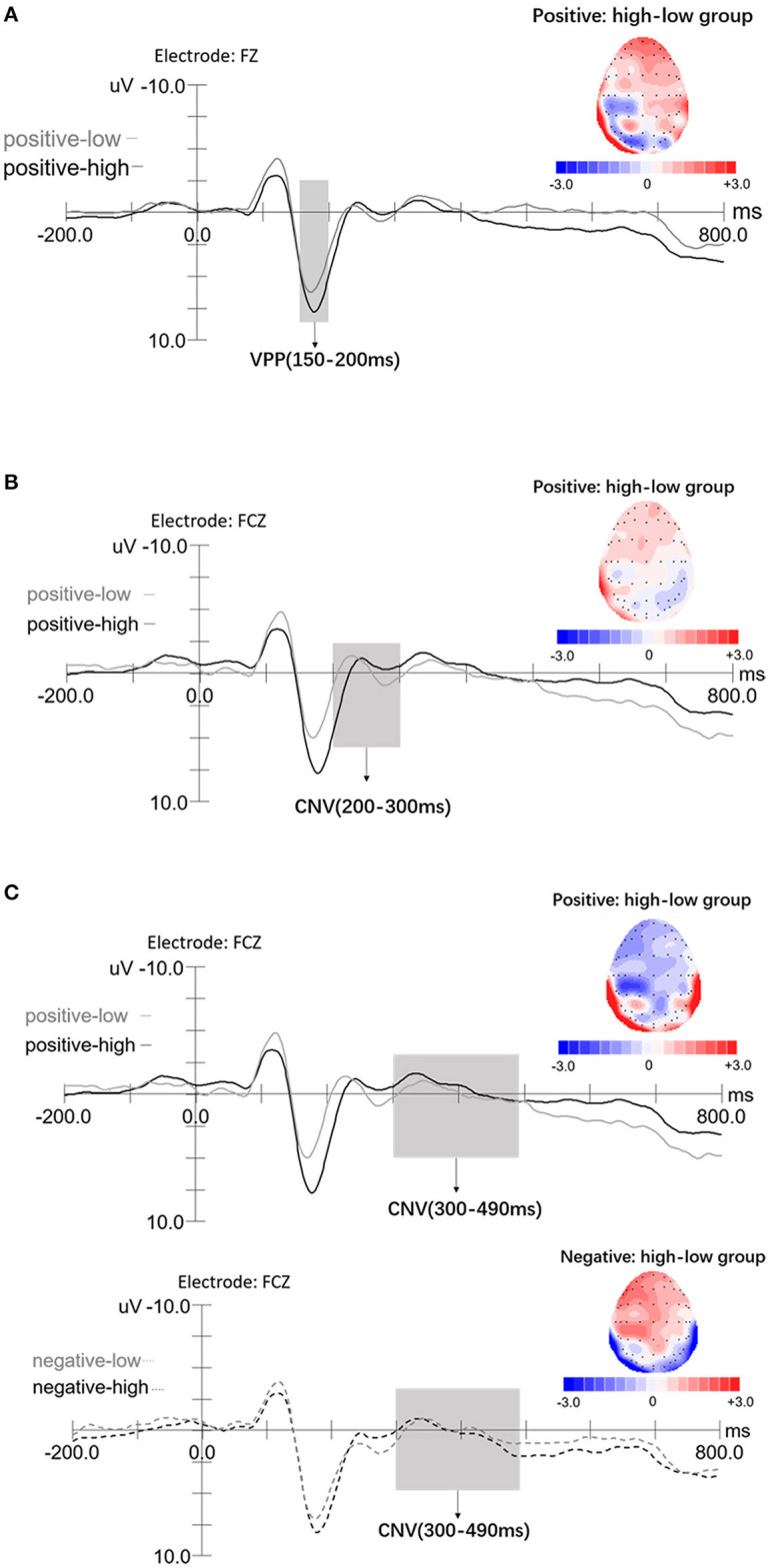
ERPs elicited by the high emotional awareness (black lines) and low awareness (gray lines) displayed for VPP (150–200 ms) and CNV (200–300, 300–490 ms) components, with −200 to 800 ms time span. The *x*-axis represents time (ms) and the *y*-axis represents amplitude (μV). **(A)** Grand-average ERP of the VPP component at the onset of the emotion pictures at Fz for an example diagram; a: topographic maps of difference waves in the positive emotion condition (high awareness group minus low awareness group). **(B)** Grand-average ERP of CNV (200–300 ms) component at the onset of the emotion pictures at frontocentral area (FCz); a: topographic maps of difference waves in the positive emotion condition (high awareness group minus low awareness group). **(C)** Grand-average ERP of CNV (300–490 ms) component at the onset of the emotion pictures at frontocentral area (FCz); a, b: topographic maps of difference waves in the positive emotion and negative emotion (high awareness group minus low awareness group), respectively.

The mixed rmANOVA with the between-factor awareness group (2: high/low) and the within-factor emotion valence (3: neutral, positive, and negative) and electrodes (3: F1, FZ, and F2) revealed a main effect of emotion valence, *F*_(2,80)_ = 26.51, *p* < 0.001, η^2^ = 0.40. Bonferroni *post hoc* test showed that the amplitudes of the negative emotion (7.21 ± 0.50 μV) were larger than that of the positive emotion condition (6.42 ± 0.47 μV), *p* = 0.01, and that of the positive emotion condition was larger than that of the neutral condition (5.80 ± 0.52 μV), *p* = 0.01. The electrode factor also resulted significant, *F*_(2,80)_ = 12.72, *p* < 0.001, η^2^ = 0.24; VPP amplitudes at Fz (6.68 ± 0.51 μV) were larger than F1 (6.39 ± 0.47 μV) and F2 (6.37 ± 0.47 μV; *ps* < 0.001), whereas F1 and F2 showed no significant difference (*p* =1.00). The interaction between emotional awareness and emotion valence was significant, *F*_(2,80)_ = 5.50, *p* = 0.008, η^2^ =0.12. Simple-effect analysis showed that in the positive emotion condition, VPP amplitudes in the high awareness group (7.24 ± 0.66 μV) were larger than in the low group (5.60 ± 0.66 μV), *F*_(1,40)_ = 3.06, *p* = 0.048, η^2^ = 0.07. There were no significant differences between the high and low awareness groups in the neutral or negative emotion conditions (*ps* ≥ 0.12). The interaction between emotional awareness and electrodes was significant, *F*_(2,80)_ = 6.81, *p* = 0.003, η^2^ = 0.15. Simple-effect analysis showed that for the low group, VPP amplitudes at Fz (5.98 ± 0.71 μV) were larger than at F2 (5.68 ± 0.67 μV), *p* = 0.002; however, no other significance differences were observed. For the high awareness group, VPP amplitudes at Fz (7.38 ± 0.71 μV) were larger than F1 (6.86 ± 0.67 μV), *p* < 0.001; VPP amplitudes at Fz were larger than F2 (7.05 ± 0.67 μV), *p* = 0.001. No other significant main or interactive effects were observed (*p*s ≥ 0.09). As shown in Figure 3A, we selected Fz as the example diagram to show the groups' difference (in positive emotion) of VPP components, a: for the topographic map.

#### CNV

Average amplitudes of CNV components (FCz; 200–300 ms), measured by a mixed rmANOVA with the between-factor awareness group (2: high/low) and the within-factor emotion valence (3: neutral, positive, and negative), revealed a main effect of emotion valence, *F*_(2,80)_ = 30.98, *p* < 0.001, η^2^ = 0.44. *Post hoc* analyses using Bonferroni correction showed that the CNV amplitudes for negative emotion (1.88 ± 0.59 μV) were larger than in the positive emotion condition (1.07 ± 0.58 μV), *p* = 0.001, whereas the CNV amplitudes (200–300 ms) in the positive emotion condition were larger than in the neutral condition (0.26 ± 0.53 μV), *p* < 0.001. The interaction between emotion valence and awareness group was significant, *F*_(2,80)_ = 11.25, *p* < 0.001, η^2^ = 0.22. Simple-effect analysis showed that in the positive emotion condition, the high awareness group (2.33 ± 0.82 μV) elicited larger CNV amplitudes (200–300 ms), compared with the low awareness group (−0.19 ± 0.82 μV), *F* (1, 40) = 11.25, *p* = 0.035, η^2^ = 0.11. The high and low awareness groups showed no differences in the neutral or negative emotion conditions (*p*s ≥ 0.34); moreover, no main effects of awareness group were observed (*p* = 0.21). As shown in Figure 3B, we selected FCz to show the groups' difference (in positive emotion) of CNV components (200–300 ms), a: for the topographic map.

A mixed rmANOVA with the between-factor awareness group (2: high/low) and the within-factor emotion valence (3: neutral, positive, negative) and CNV components (FCz; 300–490 ms) revealed a main effect of emotion valence, *F*_(2,80)_ = 90.05, *p* < 0.001, η^2^ = 0.69. *Post hoc* analyses showed that CNV amplitudes (300–490 ms) in the negative emotion condition (0.66 ± 0.49 μV) were larger than in the positive (−0.32 ± 0.48 μV) or neutral conditions (−0.26 ± 0.49 μV), *ps* < 0.001. The interaction between emotion valence and awareness groups was significant, *F*_(2,80)_ = 14.61, *p* < 0.001, η^2^ = 0.27. Simple-effect analysis showed that in the positive emotion condition, CNV amplitudes (300–490 ms) of the high awareness group (−1.23 ± 0.68 μV) were larger than that of the low awareness group (0.51 ± 0.68 μV), *F*_(1,40)_ = 2.87, *p* = 0.038, η^2^ = 0.07. In the negative emotion condition, the high awareness group (1.19 ± 0.66 μV) elicited larger CNV amplitudes (300–490 ms), compared with the low awareness group (0.13 ± 0.66 μV), *F*_(1,40)_ =11.87, *p* = 0.049, η^2^ = 0.06. In addition, the high and low awareness groups showed no difference in the neutral emotion condition (*ps* = 0.93), and the awareness group had no main effect (*ps* = 0.33). As shown in Figure 3C, we selected FCz to show the groups' differences (in positive/negative emotion) of CNV components (300–490 ms), a, b: for the topographic map in positive emotion and negative emotion, respectively.

## Discussion

This study used a temporal generalization paradigm to explore the relationship between emotional awareness and time perception, through behavior and ERP components. The behavior results revealed that the time overestimation of the high and low awareness groups had no difference in the neutral and negative emotion conditions, but showed significant difference in the positive emotion condition. The ERP results showed that in the positive emotion condition, the high awareness group elicited larger VPP amplitudes, compared with that of the low awareness group. The high and low awareness groups had no difference in the neutral and negative emotion conditions. The CNV (200–300, 300–490 ms) results showed that in the positive emotion condition, the high awareness group elicited larger amplitudes, compared with that of the low awareness group. However, for the negative emotion condition, the high awareness group elicited larger CNV (300–490 ms) amplitudes, compared with that of the low awareness group. Whereas, the high and low reported no significant differences in the neutral emotion condition of the CNV components, our results indicated that the high and low awareness groups had a time overestimation difference on emotional pictures.

### Behavioral Performance

Our behavioral results show a time overestimation difference in the emotion (mainly positive emotion) conditions between the high and low awareness groups. These findings are consistent with our hypothesis and can be explained by the arousal-based time perception hypothesis. Arousal and attention are the main factors affecting time perception (Angrilli et al., 1997; Droit-Volet et al., 2013; Schwarz et al., 2013; Van and Balsam, 2014; Fayolle et al., 2015); arousal-based time perception causes time overestimation, whereas attention-based time perception leads to underestimation. These time distortions can be attributed to the internal clock models (Chambon et al., 2005; Tipples, 2008; Droit-Volet et al., 2010, 2013). Arousal-based time perception has revealed that higher emotional arousal increases the speed of the pacemaker quickly, accumulating more pulses, resulting in the overestimation of time duration (Treisman, 1963; Schwarz et al., 2013; Droit-Volet and Gil, 2016; Cui et al., 2018). Moreover, the attention-based time distortion results showed that online accumulation of temporal pulses during the timed stimulus is compromised when we pay less attention to time, resulting in the underestimation of time duration (Lejeune, 1998; Macar, 2002; Coull et al., 2004; Droit-Volet and Meck, 2007). The time overestimation results showed differences between the high and low awareness groups in the emotion condition, whereas the underestimation results showed no difference between the groups. The results are consistent with previous findings suggesting that, within 2 s, emotional arousal played the main role in time perception (Angrilli et al., 1997; Gan et al., 2009a; Lake et al., 2016). In the positive emotion condition, the high emotional awareness group showed higher physiological arousal, compared with the low awareness group (Zhang et al., 2016). This study extended prior findings by examining the relationship between arousal and time perception from the perspective of individual differences.

### ERP

In accordance with our expectations, the VPP amplitude of the high awareness group was significantly larger than that of the low awareness group in the emotion (mainly positive emotion) condition. The VPP component is an index for distinguishing emotional and neutral expression; the amplitude of the emotional stimuli is larger than that of neutral stimuli (Vogel et al., 1998; Luo et al., 2010; Wang et al., 2015). The VPP results may suggest that the high awareness group (compared with the low awareness group) has an advantage in differentiating between positive expression and neutral expression. This is supported by prior findings where the high awareness group easily recognized positive emotion, whereas the low awareness group reported some difficulties (Wang et al., 2015). Therefore, the VPP results may provide electrophysiological evidence for the relationship between emotional awareness and time perception. High emotional awareness has been found to result in higher emotional arousal (Zhang et al., 2016), and that participants with high arousal tended toward larger time overestimation (Effron et al., 2006; Chambon et al., 2008; Droit-Volet et al., 2010).

Consistent with our hypotheses, the results of CNV components (200–300, 300–400 ms) showed that in the emotion (mainly positive) condition, the amplitudes of the high awareness group were larger than that of the low awareness group. This may suggest that CNV amplitude was not only an index of emotional arousal, but also of the accumulation mechanism and time overestimation, especially at the FCz electrode site (Olofsson et al., 2008; Klorman and Ryan, 2010; Mella et al., 2011; Pouthas et al., 2015). The CNV results suggest that the high awareness group may have a higher physiological arousal and timing accumulation, compared with the low awareness group in the positive emotion condition. This is partially consistent with previous findings that the high awareness group had a higher physiological arousal in the emotion condition, compared with the low awareness group (Zhang et al., 2016), and that higher arousal led to more time overestimation (Chambon et al., 2008; Droit-Volet et al., 2010). The CNV results extend existing evidence using electrophysiological techniques.

It should be noted that both behavioral and ERP results showed the influencing effects of emotional awareness on time perception in the positive emotion condition. However, in the negative emotional condition, we found almost no such effects. One possible reason may be that the negative emotional material combines images of fear, anger, and sadness. The complexity of negative emotional materials may confound the effect of emotional awareness on time perception. This is supported by research demonstrating that, at the same emotional arousal level, fear images are more likely to induce an overestimation of time than sad images (Droit-Volet and Gil, 2016). Future studies should consider using purer experimental materials to investigate this effect.

## Conclusion

This study aimed to explore the neural mechanism of the influence of high and low emotional awareness on time perception. This study provides evidence that in the positive emotion condition, participants with high emotional awareness tend toward larger time overestimations, compared with those with low emotional awareness.

## Data Availability Statement

The raw data supporting the conclusions of this article will be made available by the authors, without undue reservation.

## Ethics Statement

The studies involving human participants were reviewed and approved by Ethical Committee of the School of Psychology at Shanghai Normal University. The patients/participants provided their written informed consent to participate in this study.

## Author Contributions

JM contributed to conceptualization, acquisition, collection, analysis, interpretation, and drafting. XL contributed to conceptualization, interpretation, and revision of the work. JL contributed to supervision and validation. All authors contributed to the article and approved the submitted version.

## Funding

This project was granted financial support from the Humanities and Social Sciences Research Youth Fund from Ministry of Education of China (grant number 20YJC190011).

## Conflict of Interest

The authors declare that the research was conducted in the absence of any commercial or financial relationships that could be construed as a potential conflict of interest.

## Publisher's Note

All claims expressed in this article are solely those of the authors and do not necessarily represent those of their affiliated organizations, or those of the publisher, the editors and the reviewers. Any product that may be evaluated in this article, or claim that may be made by its manufacturer, is not guaranteed or endorsed by the publisher.
